# Occupational cancer burden: the contribution of exposure to process‐generated substances at the workplace

**DOI:** 10.1002/1878-0261.12925

**Published:** 2021-02-17

**Authors:** Ann Olsson, Hans Kromhout

**Affiliations:** ^1^ International Agency for Research in Cancer (IARC) World Health Organization (WHO) Lyon France; ^2^ Institute for Risk Assessment Sciences Utrecht University The Netherlands

**Keywords:** diesel engine exhaust, occupational cancer burden, occupational exposures, process‐generated substances, respirable crystalline silica, welding fumes, wood dust

## Abstract

Respirable crystalline silica in mineral dust, wood dust, diesel engine exhaust emissions and welding fumes are among the most common process‐generated substances to which millions of workers are exposed daily. The composition of process‐generated substances can vary substantially, depending on the parameters of the underlying processes; for example, the composition and intensity of diesel motor emissions differs among the various generations of diesel engines and working environments (e.g. surface or underground mining). We illustrate how common these occupational exposures are and discuss challenges in estimating their global prevalence and their contribution to the burden of occupational cancer. Estimates of the number and proportion of workers exposed in most countries and on a global scale are generally scarce. A remarkable exception is based on the proactive bottom‐up estimates generated within the European Network for Silica. Actions to reduce exposures and research to fill gaps in knowledge adapted to local settings are warranted to mitigate the occupational cancer burden, especially in under‐researched settings including low‐ and middle‐income countries.

## Graphical abstract

Occupational exposure to respirable crystalline silica, diesel engine exhaust emissions and welding fumes are widespread risk factors for lung cancer and account for approximately half of the occupational lung cancer burden. If employers succeed in controlling workplace exposures to these process‐generated substances, the fraction of lung cancers attributable to occupational exposures could be reduced dramatically.

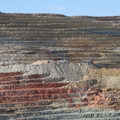

**Abbreviations**
AWES: Australian Work Exposures Study
CAREX: CARcinogen EXposure, international information system on occupational exposure to carcinogens
CEE: Central and Eastern Europe
ECHA: European Chemicals Agency
ECRHS: European Community Respiratory Health Survey
EU: European Union
IARC: International Agency for Research in Cancer
IMA‐DMP: European Industrial Minerals Association Dust Monitoring Program
NEPSI: European Network on Silica
NIOSH: National Institute for Occupational Safety and Health
NMSC: nonmelanoma skin cancer
OccIDEAS: Occupational Integrated Database Exposure Assessment System
OSHA: Occupational Safety and Health Administration
PAF: population attributable fraction
RCS: respirable crystalline silica
REACH: Registration, Evaluation, Authorisation and Restriction of Chemicals
REL: recommended exposure limit
REP: risk exposure periods
RERI: relative excess risk due to interaction
SCOEL: Scientific Committee on Occupational Exposure Limits

## 1 The burden of cancer related to occupational exposures

**Fig. 1:**
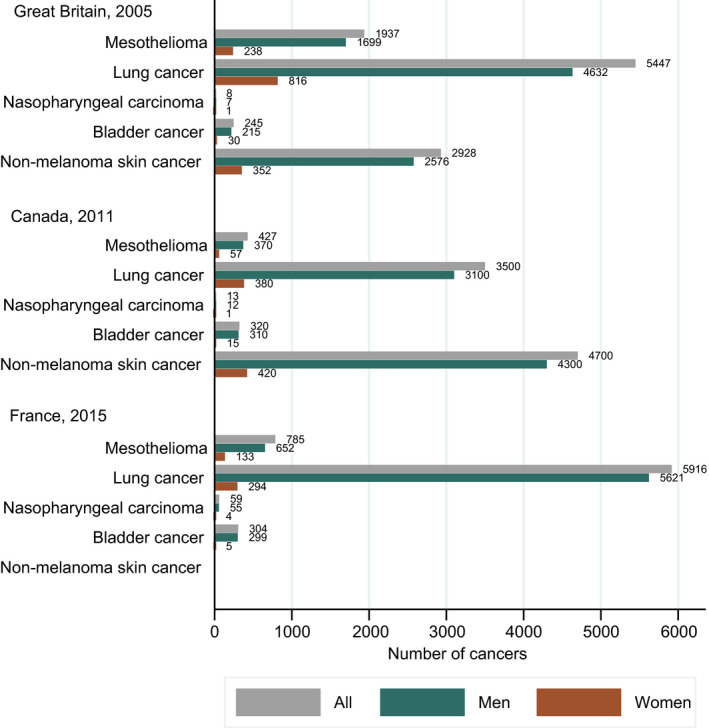
Number of cancer cases attributable to occupational exposures at selected cancer sites in Great Britain [1], Canada [2] and France [3] in the total population and by gender.

Cancers caused wholly or partly by exposure to carcinogenic agents at work or by circumstances at work can be referred to as occupational cancers. The most frequent occupational cancers are lung cancer, mesothelioma and bladder cancer. Figure 1 shows the numbers of cancers attributable to occupational exposures in the total population and stratified by sex in three nationwide studies in Great Britain, Canada and France [1, 2, 3]. Occupational cancers are preventable by eliminating hazardous substances or enhancing the protection of workers and reducing exposures.

In epidemiology, the ‘population attributable fraction’ (PAF) is estimated to assess the public health impact of specific risk factors and to rank them. The PAF, usually presented as a percentage, represents the estimated proportion of cases that would not have occurred if the exposure had not been present. The PAF is directly determined by the magnitude of risk associated with the exposure and the prevalence and level of the exposure in the working population [4].

**Fig. 2:**
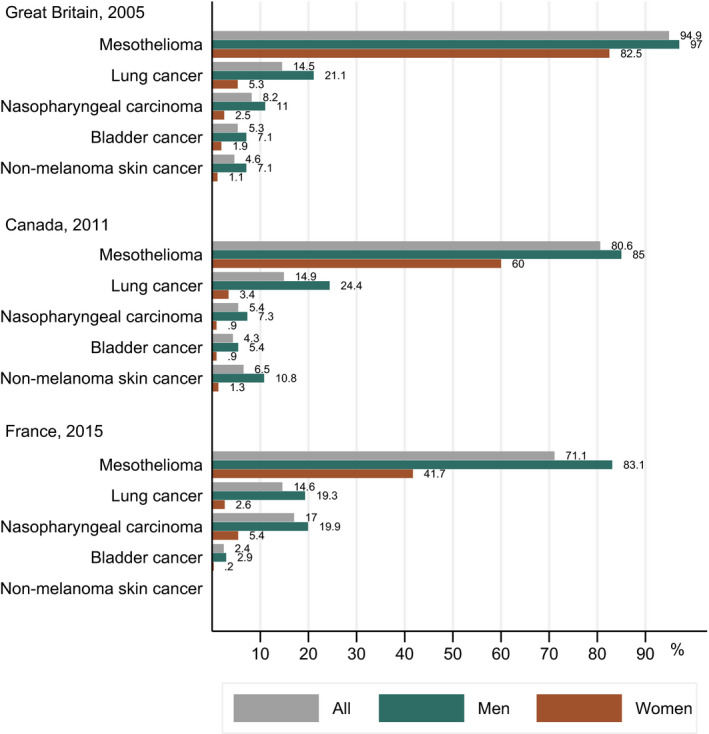
Cancer burden (%) attributable to occupational exposures at selected cancer sites, in Great Britain [1], Canada [2] and France [3] in the total population and by gender.

The overall cancer burden attributable to occupational exposures has most often been estimated between 2% and 5% since the 1980s [1, 2, 3, 5]. Figure 2 shows the PAFs for occupational exposures (total and stratified by sex) for selected cancers in Great Britain, Canada and France [1, 2, 3]. Mesothelioma is mainly associated with exposure to asbestos [6]. Lung cancer is associated with many occupational exposures including asbestos, crystalline silica, diesel engine exhaust and welding fume [6, 7, 8]. Nasopharyngeal cancer is associated with wood dust and formaldehyde [6, 9]. Urinary bladder cancer is associated with exposure to aromatic amines, diesel engine exhaust and exposures in painting and the rubber industry [9]. Nonmelanoma skin cancer (NMSC) is associated with exposure to solar radiation, coal tars and pitches, mineral oils and arsenic [6, 10]. The figure shows a marked difference in PAFs by cancer site with mesothelioma having the highest occupational PAF, followed by lung cancer, nasopharyngeal, bladder and NMSC. There are also large differences in occupational PAFs by sex, with generally larger PAFs among men. Men are indeed 4–5 times more likely to work in primary production jobs with higher occupational exposures compared with women [11, 12]. Finally, it is noticeable that not all cancer types are included in all studies; for example, NMSC is not included in the French study.

PAFs are not easily comparable across studies due to differences in the selection of exposure‐cancer combinations, the use of varying criteria to define prevalence and level of exposures encountered, differences in the selection of risk estimates associated with the exposures, etc. For example, the French study included only IARC Group 1 substances (*n* = 25), the study from Great Britain included in addition Group 2A substances (*n* = 41) and the Canadian study included 44 exposures whereof two Group 2A (creosotes and night shift work) [1, 2, 3]. The French project was studying the burden of cancer attributable to all modifiable risk factors and therefore assigned ‘second‐hand smoke’ to ‘cancers attributable to tobacco smoking’ rather than occupational exposures. Moreover, solar radiation was considered as a risk factor for cutaneous melanoma attributable to solar ultraviolet radiation, together with the use of sunbeds. NMSCs were not included in the French study or the Global Burden of Disease project, possibly because NMSCs often are not systematically registered and therefore may be severely under‐reported [13, 14, 15]. Other reasons to exclude exposures or exposure‐cancer combinations from the estimation are the lack of exposure prevalence data, or that exposures are no longer relevant or thought to be present (e.g. after asbestos use bans) [16].

The methodology for estimating PAFs has evolved to account for relevant risk exposure periods (REP) defined by the cancer latency for solid tumours (0–50 years) and for haematopoietic cancers (0–20 years), workers turnover (which might differ considerable between industries and especially over time) and proportion of workers exposed to high‐ *versus* low levels of exposure (however without a clear definition of high and low levels of exposure). Nonetheless, study bias and uncertainty are present. The choice of risk estimates and the employment turnover was identified as the largest contributors to the occupational PAF estimates in the British study [17].

Smoking patterns among workers should be incorporated in the methodology for estimating of the occupational cancer burden in the future. The reason is that many occupational exposures confer stronger effects among smokers. In the SYNERGY project – a large pooled analysis of case–control studies on interactions of smoking and occupational exposure risks relating to lung cancer incidence – an additive scale was used to calculate the relative excess risk due to interaction (RERI) by fitting linear odds ratio (OR) models [18]. The RERI measures the extent to which the effect of both exposures (e.g. smoking and having worked as a painter) combined exceeds the sum of the effects of each considered separately, and a RERI > 0 indicates a positive additive interaction, where the effect of both exposures together exceeds the sum of the two exposures considered separately. Given that smoking is a strong risk factor for lung cancer, this marked difference in smoking habits between men and women especially in the past may, at least partly, explain why we often see less effect of occupational exposures in exposed women than in exposed men [18, 19, 20, 21]. A recent paper by Kulhánová *et al*. [22] shows that the tobacco‐related cancer burden in Europe differs across countries and genders; the largest and the lowest PAF due to smoking in males occurred in Eastern Europe (35% of all cancer cases) and Northern Europe (21%), while among women this pattern was reversed (16% in Northern Europe and 6% in Eastern. Consequently, it would be beneficial to account for smoking patterns at the relevant period by sex in each country, when estimating the occupational cancer burden.

In this review, we discuss the contribution of process‐generated substances to the occupational cancer burden, with a focus on occupational exposure to crystalline silica, wood dust, diesel engine exhaust and welding fumes. Our aim is to illustrate how common these exposures have been and still remain, to a large extent, in the working environment, and to discuss their contribution to the global occupational cancer burden. We also address the challenges in estimating the global prevalence of these occupational exposures and the subsequent burden of occupational cancer.

## 2 Process‐generated substances

Process‐generated substances are generated as emissions from combustion or heating processes, abrasion and other processes that physically or chemically modify or degrade the starting material(s) and are, thus, present in various workplaces. Respirable crystalline silica in mineral dust, wood dust, diesel engine exhaust emissions, welding fumes, flour dust, bitumen fumes and rubber curing fumes are some examples of common process‐generated substances affecting millions of workers daily. While a clear definition is not readily available, these process‐generated substances often exist as mixtures and can be of chemical or biological nature. They are by their nature more complex than single chemical substances or single biological species, and they are not seen as manufactured products that can be traded and tracked along a supply chain. Therefore in the EU, these substances are not regulated by the Registration, Evaluation, Authorisation and Restriction of Chemicals (REACH) of the European Union (EU) REACH regulation in the EU [23], but they are part of the Carcinogens and Mutagens Directive 2004/37/EC [24]. Since 2019, the Risk Assessment Committee of the European Chemicals Agency (ECHA) provides scientific opinions on occupational exposure limits after it took over this responsibility from the Commission's Scientific Committee on Occupational Exposure Limits (SCOEL).

The composition of a process‐generated substance can vary substantially, depending on the parameters of the underlying process. For instance: differences in recipes for rubber compounding may produce vulcanization or curing fumes that differ dramatically not only in composition, but also in levels of the individual chemicals present in these fumes [25]. Similarly, the composition of organic dusts can differ dramatically when working with organic material with different moisture content [26]. For process‐generated substances such as diesel motor emissions, the composition and intensity will differ among the various generations of diesel engines and environments where human exposure occurs (e.g. surface or underground mining) [27].

For many process‐generated substances, exposed populations are considerably larger when compared to single (chemical) substances. Kauppinen *et al*. (2000) generated in a Europe Against Cancer project called CAREX (CARcinogen EXposure) the number of workers exposed in the then 15 EU member states in the early 1990s. The CAREX project covered all agents, groups of agents and process‐generated substances which were considered by the International Agency for Research on Cancer (IARC) as known or suspected carcinogenic agents as of February 1995 [28]. The prevalence estimates from the CAREX paper indicated that the number of workers exposed to certain process‐generated substances make up for the majority of exposures to known or suspected carcinogenic chemical agents [29]. The four most prevalent exposures to substances described within the CAREX project included the following process‐generated substances: environmental tobacco smoke > 75% of working time, crystalline silica, diesel engine exhaust and wood dust. These four process‐generated substances resulted in an estimated total of slightly more than 16 million exposed workers in 15 member states, which represented 55% of the total number of workers exposed to 80 known or suspected carcinogenic substances considered.

The CAREX approach has consequently been applied and adapted to national circumstances by experts in industrial hygiene, other experts and data sources in individual countries including Costa Rica, Estonia, Czech Republic, Latvia and Lithuania [30, 31, 32]. In Costa Rica, CAREX was renamed TICAREX and included self‐employed persons and working family members older than 12 years to reflect the national working situation more accurately. The top‐10 substances in TICAREX were largely overlapping with the original CAREX, but it also provided separate numbers for men and women [32]. In the Baltic countries, there were some differences noted such as that exposure to wood dust being more prevalent in Estonia due to large wood and furniture industries, while for Lithuania, the prevalence of benzene exposure was more prevalent due to the presence of oil refineries [30].

A more recent and refined carcinogen surveillance project modelled on the original European CAREX, called CAREX Canada, showed similar prevalence of carcinogen exposures for the year 2006 [33]. The process‐generated substances diesel engine exhaust, crystalline silica, polycyclic aromatic hydrocarbons (PAHs) and wood dust were again located at the top of the list of 40 known or suspected carcinogenic chemical agents considered, and as in Europe, these agents represented half of the workers' exposures.

A study in Quebec made use of CAREX Canada but refined the estimates by including additional workplace monitoring data, research projects, a population survey and radiation protection data [34]. The European and the Costa Rican CAREX methodology was applied with modifications in Nicaragua and Panama and included in addition relevant pesticides. Population censuses provided industry‐ and sex‐specific workforce numbers and experts from governmental agencies, workers' organizations and employers' representatives estimated activity‐ and sex‐specific proportions of exposed workers [35].

The SHEcan project, sponsored by EC DG Employment in Luxembourg and carried out between 2009 and 2011, investigated the socioeconomic, health and environmental impact associated with a range of policy options for amendments to Directive 2004/37/EC (Carcinogens or Mutagens at work) [36]. The purpose of the assessment was to enable the European Commission to initiate informed discussions with stakeholders about potential impacts of changes in legislation. The SHEcan reports included estimates of the number of workers exposed [36].

The relatively recent Australian Work Exposures Study (AWES) conducted a cross‐sectional survey including about 5000 respondents (53% response fraction) from a random sample of the population. Data were collected by trained interviewers using a computer‐assisted telephone interview. An automated expert assessment method (OccIDEAS) assigned exposure to carcinogens based on the general job information and when justified job‐specific modules [37]. The prevalence figures were thereafter extrapolated to the Australian working population by sex. OccIDEAS allows modifying definitions of exposure, for example to define ‘substantial exposure’ of a substance resulting in an alternative prevalence of exposure [38].

Table 1 provides an overview of estimates of number of workers (men and women) and percentages of the workforce exposed to crystalline silica, wood dust, diesel engine exhaust and welding fumes in different regions/countries in different periods.

**Table 1:** Overview of studies and other resources estimating number of workers (men and women) and percentages exposed to respirable crystalline silica, wood dust, diesel engine exhaust and welding fumes.

Study [Reference]	Location	Year(s)	Number of workers and percentages (%) of exposed to selected process‐generated substances				Total working population
			Respirable crystalline silica	Wood dust	Diesel engine exhaust	Welding fumes	
CAREX [29]	EU‐15	1990–1993	3 200 000 (2.3)	2 600 000 (1.9)	3 000 000 (2.2)	‐	139 000 000
Occupational cancer in Britain [56]	UK	1990–1993, 1979	564 787 (2.0)	433 834 (1.5)	473 062 (1.6)	172 418 (welders)	28 768 000^a^
CAREX [30]	Estonia	1997	19 000 (3.1)	34 000 (5.5)	21 000 (3.4)		620 689
	Latvia	1997	19 000 (2.0)	35 000 (3.8)	20 000 (2.2)		928 571
	Lithuania	1997	40 000 (2.4)	47 000 (2.8)	37 000 (2.2)		1 678 571
	Czech Republic	1997	170 000 (3.4)	180 000 (3.6)	130 000 (2.6)		5 000 000
TICAREX [32]	Costa Rica	2000	27 100 (2.1)	32 200 (2.5)	278 000 (21.4)		1 300 000
CAREX/SALTRA program [35]	Panama	2006	66 274 (6.9)	22 091 (2.3)	258 374 (26.9)		960 500
	Nicaragua	2007	31 213 (1.5)	47 860 (2.3)	407 856 (19.6)		2 080 899
CAREX Canada [33]	Canada	2006	382 000 (2.3)	338 000 (2.0)	781 000 (4.6)		16 800 000
CAREX Canada [34]	Quebec	2006	57 600 (1.6)	101 600 (2.8)	152 900 (4.2)		3 600 000
Australian Work Exposures Study (AWES) [37]	Australia	2011–2012	586 900 (6.5)	478 320 (5.3)	1 599 700 (17.9)		8 933 000
SHECAN (http://www.occupationalcancer.eu/projresults.html)	EU‐25	2006	5 300 000 (2.6)	Hardwood 3 000 000 (1.4)	3 600 000 (1.7)		206 700 000^b^
IARC Monographs on the Evaluation of Carcinogenic Risks to Humans Volume 118. Welding, Molybdenum Trioxide and Indium Tin Oxide [51]	Worldwide	2017				110 000 000 (3)	3 500 000 000

^a^ UK Commission for employment and skills. Working Futures 2010–2020: Main Report. August 2012, page 83.

^b^ EUROSTAT Euro‐indicators 37/2007 14 March 2007.

### 2.1 Respirable crystalline silica

From Table 1, it is clear that the prevalence of occupational exposure to respirable crystalline silica (RCS) is estimated to be between 2% and 3% of the working population, but estimates for Panama, Nicaragua and Australia are much higher at 6–7%. This might be the consequence of different methodological approaches. In Europe, fifteen industry sector organizations and their counterpart trade union federations negotiated a multisectoral social dialogue agreement on (exposure to) crystalline silica that was signed by all parties in October 2006 [39]. This unique agreement resulted among others in a bottom‐up exercise of reporting numbers of workers exposed to crystalline silica at 6200 sites (85% of the total number of sites) of 19 industrial sectors. In the 2018 report of the European Network on Silica (NEPSI), it was estimated that 180 000 workers were exposed to RCS [40]. This is considerably lower than the 320 000 estimated by Kauppinen *et al*. [29] for the early 1990s, but is most likely due to the large construction and agricultural sectors not being part of NEPSI where exposure to respirable crystalline silica does occur [41, 42].

Evidence of long‐term trends in levels of RCS exposure is abundant. Peters *et al*. [43] reported an overall downward time trend in RCS exposure levels in Europe of −6% per year across all industries over a time period covering 1976–2009. Analyses of the European Industrial Minerals Association Dust Monitoring Program (IMA‐DMP) that started in 2000 showed overall downward temporal trends of −9.0% and −3.9% per year for, respectively, respirable dust and respirable quartz. No downward trends and even a slight increase were seen within the IMA‐DMP during the most recent global economic crisis between 2008 and 2012. After this period, exposure concentrations started to decline again [44].

The National Institute for Occupational Safety and Health in the United States (NIOSH) estimated the numbers and percentages of workers exposed to respirable crystalline silica at levels of at least 1, 2, 5 and 10 times the NIOSH recommended exposure limit (REL 50 µg·m^−3^), based on the Occupational Safety and Health Administration (OSHA)'s compliance inspection sampling data from 1979 to 2015. Approximately 100 000 workers were exposed to crystalline silica above the REL, and most (~ 80%) worked in the construction industry [45].

### 2.2 Wood dust

From Table 1, the estimates of prevalence of exposure to wood dust vary between 1.4% and 5.5% of the workforce. Local differences in construction and availability of wood as construction material as well as difference in definition (only hardwood exposure considered in some estimates) will be underlying these considerable differences in prevalence estimates.

Quite a considerable number of studies on long‐term trends in wood dust exposure concentrations exist. Galea *et al*. [46] reported an annual decrease of −8% during over a 20‐year period (1985–2005) in wood‐treating industries. An almost similar annual trend of −7% was derived from two cross‐sectional studies in the furniture industry in Denmark over the turn of the century (1997–2004) [47]. Long‐term annual trends of −11% in wood dust exposure concentrations were reported for different industries based on data in OSHA's Integrated Management Information System (1979–1997). A recent study from Sweden in the wood pellet production showed an even steeper decline of −20% per annum [48].

### 2.3 Diesel engine exhaust emissions

Estimates of the number of workers exposure to diesel engine exhaust emissions vary wildly, with earlier estimates varying between 1% and 3% of the workforce, later estimates around 4–5% and most recent estimates providing unlikely extremely high prevalence estimates of 18–27%. Most likely, methods applied and definitions of what occupational exposure to diesel engine exhaust entails will be accountable for this. The authors explained that very few exposure measurements have been conducted in Nicaragua and Panama, and therefore, they relied heavily on expert judgment (*n* = 25 in each country) from relevant authorities [35].

A few long‐term trends in levels of exposure to diesel engine exhaust emissions exist. For the U.S. trucking industry, sharp declines in exposure to elemental carbon (as a marker for diesel engine exhaust emissions) were estimated which were job group specific [49]. Exposure levels differed between and within job groups as well as between types of trucking terminal, and regions of the United States. Two large studies in the mining industry also showed diverse trends and large difference between job groups and between surface and underground work [27, 50].

### 2.4 Welding fumes

Welding‐related exposures were recently classified a human carcinogen by IARC, and it was estimated by the working group during the review process that 11 million workers are exposed as (full‐time) welders, while the total number of workers (part‐time) exposed to welding fumes was estimated to be around 110 million (3% of the worldwide economically active population) [51].

Studies on long‐term trends in exposure to welding fumes are hard to find. For the European Community Respiratory Health Survey (ECRHS) II study, a welding fume algorithm was developed based on a database of 1233 welding fume personal measurements from the Netherlands collected over a 20‐year period [52, 53]. A relatively minor 2–3% annual decrease in welding fume concentrations (halving of concentrations after 20–35 years) was observed. Within the ECRHS study, cumulative exposures in Northern Europe were lower than in Southern Europe most likely as result of differences in welding techniques, available control measures and/or hours welding per day/week, since no difference was seen in average number of years welding in Northern and Southern Europe.

## 3 Conclusions

The PAF for lung cancer due to occupational exposure has been estimated to be between 18 and 25% in men and 2–6% in women, resulting in lung cancer being the most prevalent occupational cancer [1, 2, 3]. Generally, occupational exposure to asbestos is considered to be contributing the most to the occupational PAF for lung cancer, followed by occupational exposures to respirable crystalline silica, diesel engine exhaust emissions and welding fumes. Table 2 shows examples of occupational PAFs for lung cancer for the selected process‐generated substances and confirms the ranking although the PAFs vary slightly by study and study type. Together exposure to respirable crystalline silica, diesel engine exhaust emissions and welding fumes account for half of the occupational PAF for lung cancer. If employers succeed in controlling workplace exposures to process‐generated substances, the fraction of lung cancers attributable to occupational exposures would be reduced dramatically.

**Table 2:** Overview of studies estimating the occupational lung cancer burden (PAFs) related to selected process‐generated substances and the source of the estimated exposure prevalence in different types of studies.

Reference	Where, when	Population attributable fractions (%)						Source of proportion exposed
		Respirable crystalline silica		Welding fumes		Diesel engine exhaust		
		Men	Women	Men	Women	Men	Women	
Boffetta *et al*. [16]	France, 2000	0.5	0.07					SUMER 1994 survey
Olsson *et al*. [66]	CEE, 1998–2002	4.9	2.2					IARC CEE case–control study
De Matteis *et al*. [67]	Italy, 2002–2005	5.7	–					EAGLE case–control study
Rushton *et al*. 2010 [68]	GB, 2005	4.2	0.4	0.7	0.1	3.0	0.5	CAREX 1990–1993, Labour Force Survey
Labrèche *et al*. [3]	Canada, 2011	4.4	0.2	2.4	0.08	4.3	0.2	CAREX Canada
Marant Micallef *et al*. [4]	France, 2015	1.5	0.1			1.4	0.1	SUMER 2003 survey

Kauppinen and coworkers should be applauded for inclusion of suspected carcinogenic agents in the original CAREX project carried out 35 years ago, because the number of known occupational carcinogens has increased over time to 47 agents identified as known occupational carcinogens in 2017, compared with 28 in 2004 [54]. Three of the four selected process‐generated substances discussed in this paper, namely crystalline silica, diesel motor exhaust and welding fumes, were classified by IARC as lung carcinogens (Group 1) only after the creation of CAREX. Welding fume is most often excluded from studies calculating PAFs related to occupational exposures, because it was classified as carcinogenic to the lung only in 2017 and the prevalence of the occurrence of this exposure is not easy to assess. However, exposure to welding fumes will have partly overlapped with other exposures such as hexavalent chromium in several studies.

The original CAREX has inspired many consequent projects to estimate numbers and proportions of workers exposed to carcinogenic agents, and to adapt these estimates for various countries [16, 32, 33, 34, 35, 55, 56]. Nevertheless, CAREX is still being used more than 35 years after its creation, notably in the Global Burden of Disease Project [13, 57]. This may be sufficient if the objective is only to conclude that occupational carcinogens continue contributing to the global cancer burden and to justify the need for ongoing prevention and control initiatives [58]. However, if the objective is to set the departure for controlling hazardous exposures in the workplaces, it is important to conduct workplace exposure studies in more countries than what is currently done. Also, employers, worker's associations and management are more likely to ‘act on what they see’ in local, regional or national studies, and dust monitoring in itself might result already in lower exposure concentrations [59].

Despite evidence of declining exposure in European and North American workplaces [60], comprehensive studies of the effectiveness of workplace interventions for reducing hazardous exposure remain scarce [61]. Recently, Ohlander *et al*. [61] observed an improvement in the frequency and quality of intervention studies targeting exposure to chemicals and biological agents in the workplace over the last six decades and concluded that it is important to expand the evidence on (cost‐) effectiveness and transferability of interventions to reduce exposure and health effects, in order to reduce occupational ill‐health caused by these exposures.

The prevalence of process‐generated substances and others in the majority of countries including low‐ and middle‐income countries is largely unknown because few studies have been conducted locally [13, 62, 63]. A review discussing the increasing cancer burden in Africa revealed suboptimal implementation of occupational health standards notably in the informal sector, use of outdated technologies in industry and lack of awareness of potential hazards in specific employment structures may give rise to high levels of occupational exposures. Exposures in mining and exposure to pesticides in agriculture and agents arising from the mismanagement of hazardous waste from local, industrial and transboundary sources are of particular concern [64, 65].

Process‐generated substances are by far the most prominent and prevalent occupational exposures to substances even today in Europe, Canada, Australia and a few other countries, where systematic research has been done to estimate the prevalence of occupational exposure. Unfortunately, due to this limited insight, precise estimates of the number of workers exposed (on a global scale) and turnover rates in global workforces are generally not available, and therefore, the estimates of the global burden of cancer due to these exposures will remain rather imprecise and will either overestimate or (more likely) underestimate the importance of carcinogenic exposure in the workplace. Actions to reduce exposures and research to fill gaps in knowledge adapted to local settings are warranted to mitigate the occupational cancer burden, especially in low‐ and middle‐income countries.

## Conflict of interest

The authors declare no conflict of interest.

## Author contributions

AO and HK conceived the project, AO and HK acquired the data for the tables and figures, AO and HK drafted separate parts of the paper and thereafter agreed on the interpretation and final version.

## Disclaimer

Where authors are identified as personnel of the International Agency for Research on Cancer/World Health Organization, the authors alone are responsible for the views expressed in this article and they do not necessarily represent the decisions, policy or views of the International Agency for Research on Cancer /World Health Organization.
